# Immunomodulatory properties of characellide A on human peripheral blood mononuclear cells

**DOI:** 10.1007/s10787-021-00836-5

**Published:** 2021-07-09

**Authors:** Simone Marcella, Sam Afoullouss, Olivier P. Thomas, A. Louise Allcock, Paul V. Murphy, Stefania Loffredo

**Affiliations:** 1 Department of Translational Medical Sciences, Center for Basic and Clinical Immunology Research (CISI), WAO Center of Excellence, University of Naples Federico II, Via S. Pansini 5, 80131 Naples, Italy; 2 Marine Biodiscovery, School of Chemistry, Ryan Institute, National University of Ireland Galway (NUI Galway), University Road, Galway, H91TK33 Ireland; 3 Zoology Department, School of Natural Sciences, Ryan Institute, National University of Ireland Galway (NUI Galway), University Road, Galway, H91TK33 Ireland; 4 School of Chemistry, National University of Ireland Galway, University Road, Galway, H91 TK33 Ireland; 5 Institute of Experimental Endocrinology and Oncology “G. Salvatore”, National Research Council, Naples, Italy

**Keywords:** IL-6, TNF-α, Simplexide, CD1d, LPS

## Abstract

Marine sponges and their associated microbiota are multicellular animals known to produce metabolites with interesting pharmacological properties playing a pivotal role against a plethora of pathologic disorders such as inflammation, cancer and infections. Characellide A and B belong to a novel class of glycolipopeptides isolated from the deep sea marine sponge *Characella pachastrelloides*. In this study, we have evaluated the effects of characellide A and B on cytokine and chemokine release from human peripheral blood mononuclear cells (PBMC). Characellide A induces a concentration- and time-dependent CXCL8, IL-6 and TNF-α release from PBMC. This production is mediated by the induction of gene transcription. Moreover, cytokine/chemokine release induced by characellide A from PBMC is CD1d-dependent because a CD1d antagonist, 1,2-bis(diphenylphosphino)ethane [DPPE]-polyethylene glycolmonomethylether [PEG], specifically inhibits characellide A-induced activation of PBMC. In conclusion, characellide A is a novel modulator of adaptative/innate immune responses. Further studies are needed to understand its potential pharmacological application.

## Introduction

Marine sponges are sessile multicellular animals characterized by a soft and sessile body (Anjum et al. 2016). Being unable to move and having limited physical defenses, many marine sponges have developed a sophisticated chemical defense system to protect themselves from predators and to avoid fouling by other marine organisms (Hertiani et al. 2010; Thomas et al. 2010). Given their ability to produce small metabolites, marine sponges have been the subject of much research which has identified thousands of different natural products including 300 novel compounds with interesting pharmacological features (Faulkner 2000, 2001, 2002; Sipkema et al. 2005; Blunt et al. 2006; Mehbub et al. 2014). Interestingly, some of the sponge-derived metabolites have been included in clinical and preclinical trials showing their efficacy as anti-inflammatory (Alcaraz and Paya 2006), antitumoral (Negi et al. 2017), immunosuppressive (Gunasekera et al. 1989), neurosuppressive (Yurchenko et al. 2020), antiviral (Sagar et al. 2010), antimalarial (Fattorusso and Taglialatela-Scafati 2009), antibiotic (Bibi et al. 2017) or antifungal (Karpinski 2019) compounds. Glycolipids represent the main components of several marine organisms and have been identified as potent activators of immune cells by binding to CD1d molecule (Wu et al. 2008). CD1d is a MHC-like presenting antigen molecule, belonging to the CD1 family, expressed on immune cells including monocytes, macrophages, dendritic cells B lymphocytes, thymocytes but not mature T cells (Exley et al. 2000; Brigl and Brenner 2004). It is also expressed in non-hematopoietic cells such as epithelial and vascular smooth muscle cells (Canchis et al. 1993). CD1d binds and presents lipid antigens to natural killer T cells with an invariant T cell receptor alpha chain (iNKT) inducing their development and activation in several pathological conditions such as infection (Skold and Behar 2003), autoimmune disorders (Mars et al. 2008), allergic responses (Lexmond et al. 2014) and tumor immunity (Ni et al. 2015). It has been demonstrated that CD1d binds α-galactosylceramide (α-GalCer) and simplexide, two glycolipids isolated from the marine sponges *Agelas mauritiana* and *Plakortis simplex* respectively (Koch et al. 2005; Loffredo et al. 2014). It was discovered that α-GalCer induced Th1 (IFN-γ) and Th2 (IL-4) cytokine production from human NKT cells in a CD1d-dependent manner (Kawano et al. 1997; Kronenberg 2005). Simplexide activated human monocytes through CD1d-producing cytokines and chemokines and showed a proinflammatory profile (Loffredo et al. 2014). Two metabolites belonging to a new class of glycolipopeptides, namely characellide A and B, were recently isolated from the deep sea marine sponge *Characella pachastrelloides*. Both characellides were first shown to inhibit reactive oxygen species (ROS) production from microglia BV-2 cell line stimulated with Lipopolysaccharide (LPS), therefore acting as anti-inflammatory mediators (Afoullouss et al. 2019). To date, there are no studies on the effects of characellides on primary immune cells. In this work, we investigated the effects of characellides A and B on human peripheral blood mononuclear cells (PBMC) and we tried to understand their mechanism of action.

## Materials and methods

### Reagents

The following were used for the experiments: l-glutamine (Sigma-Aldrich^®^ 155 St. Louis, MO, USA), antibiotic–antimycotic solution (10.000 IU/ml penicillin, 10 mg/ml streptomycin, and 25 µg/ml amphotericin B) (Lonza, Basel, CH), MEM non-essential amino acids (Microgem^®^, Naples, Italy), fetal bovine serum (FBS) (Sigma-Aldrich® 155 St. Louis, MO, USA), RPMI 1640 (Microgem^®^, Naples, Italy), Histopaque-1077 (GE Healthcare Bio-Sciences AB SE-751 84 Uppsala, Sweden), detoxified LPS (from *Escherichia coli* serotype 026:B6) (Sigma-Aldrich^®^ 155 St. Louis, MO, USA), 1,2-Bis (diphenylphosphino)ethane [DPPE]-polyethylene glycolmonomethylether [PEG] was from Avanti Polar Lipids (Alabaster, AL). Simplexide was isolated from *Plakortis simplex* at the Department of Pharmacy, University of Naples Federico II. Target-specific primers for *CXCL8*, *IL6*, *TNFα*, and *GAPDH* were designed using the Beacon Designer 3.0 (Biorad Laboratories, Milan, Italy) and produced and purified by Custom Primers (Life Technologies, Milan, Italy).

### Characellide A and B isolation

The freeze-dried sponge biomass (330 g) was extracted with MeOH-CH_2_Cl_2_ (1:1) using ultra-sonification. Crude extract (20.6 g) was fractionated into five fractions utilizing RP-C18 vacuum liquid chromatography, of decreasing polarity gradient, from H_2_O, H_2_O–MeOH (1:1), H_2_O-MeOH (1:3), MeOH and MeOH–CH_2_Cl_2_ (1:1). The MeOH fraction (628 g) was purified using RP-HPLC on a semi-preparative T3 column, 250 mm × 19 mm, 5 μm (Xselect, Waters, Milford, CT, USA), using water (A) and acetonitrile (B) as mobile phases, both acidified with 0.1% Trifluoroacetic acid (TFA), with a flow of 5 ml/min. The column was eluted with 45% B for 5 min, then a linear gradient 68% B over 12 min was performed, finally, the column was eluted at 68%B for a further 15 min. Subfraction 2 containing both characellide A and B, was collected at 6 min (*t*_R_ 6 min, 3.7 mg). This mixture was then further purified utilizing BEH amide 4.6 mm × 250 mm, 5 μm (Xbridge, Waters, Milford, CT, USA), using isocratic mobile phase conditions of water (A) and acetonitrile (B, 89%) both acidified with 0.1%TFA, with a flow rate of 1 ml/min, yielding characellide A (*t*_R_ 10 min, 2.2 mg) and B (*t*_R_ 14 min, 0.8 mg). Identification of characellide A and B was determined from a comparison of published and experimental using nuclear magnetic resonance spectroscopy (NMR) data, measured at 600 MHz equipped with a cryoprobe (Agilent DD2 NMR 600 MHz 54 mm) and High-Resolution Electrospray Ionisation Mass Spectrometry (HRESIMS) data from a qToF Agilent 6540 in ESI( +).

Characellide A: Yellowish oily solid; [α]D −30 (c 0.1, MeOH); λmax (log *s*) 225 (4.24) nm; ^1^H-NMR and ^13^C-NMR data, see Table S1. HRESIMS *m/z* 867.4705 [M + H]^+^ (867.4710 calcd. For C_41_H_67_N_6_O_14_, Δ-0.6 ppm).

Characellide B: Yellowish oily solid; [α]D −60 (c 0.1, MeOH); λmax (log *s*) 225 (4.24) nm; ^1^H-NMR and ^13^C-NMR data see Table S1. HRESIMS *m/z* 867.4700 [M + H]^+^ (867.4710 calcd. For C_41_H_67_N_6_O_14_, Δ-1.1 ppm).

### Isolation and purification of peripheral blood mononuclear cells (PBMC)

The study protocol involving the use of human blood cells was approved by the Ethical Committee of the University of Naples Federico II, and written informed consent was obtained from blood donors in accordance with the principles expressed in the Declaration of Helsinki (Protocol number 301/12). PBMC were isolated from buffy coats of healthy donors (HBsAg − , HCV − , and HIV −) obtained from a leukapheresis unit. Leukocytes were separated from erythrocytes by dextran sedimentation. PBMC were purified by Histopaque-1077 density gradient centrifugation (400×*g* for 20 min at 22 °C) and resuspended in RPMI 1640 supplemented with 5% FBS, 2 mmol/l l-glutamine, antibiotic–antimycotic solution and non-essential amino acid. Finally, the cells were used for experiments.

### Cell cultures

Characellide A and B preparations were routinely checked for LPS contamination. In particular, characellide (10 µg/ml) and LPS (100 ng/ml) were preincubated (37 °C, 30 min) with polymyxin B (50 µg/ml) (Sigma-Aldrich^®^ 155 St. Louis, MO, USA) before addition to the cells. Since characellide is not well soluble in DMSO, all experiments were done in polystyrene plates coated with various concentrations of compounds dissolved in methanol. Solvent was dried under nitrogen immediately before the addition of cells. PBMC (2 × 10^6^ cells per well) were incubated (37 °C, 5% CO_2_, 16 h) in 48-well polystyrene plates precoated with methanol alone or increased concentrations of characellide A and B (0.3–10 µg/ml) or simplexide (10 µg/ml). PBMC (2 × 10^6^ cells per well) were preincubated (37 °C, 5% CO_2_, 30 min) with increasing concentrations of DPPE-PEG (0.05–10 µg/ml) and then stimulated (37 °C, 5% CO_2_, 16 h) with LPS (100 ng/ml), simplexide (10 µg/ml) and characellide A (10 µg/ml). At the end of the experiments, the supernatants were removed, centrifuged (300×*g*, 4 °C, 5 min) and stored at  − 80 °C for subsequent determination of CXCL8, IL-6, TNF-α and IL-10 release. The kinetics of CXCL8, IL-6 and TNF-α release and mRNA expression in PBMC were also performed. Therefore cells (2 × 10^6^ cells per well) were incubated (37 °C, 5% CO_2_) at different times (2, 4, 8, 14, 18 and 24 h) with characellide A (10 µg/ml) and supernatants were collected, centrifuged (300×*g*, 4 °C, 5 min) and stored at  − 80 °C for subsequent determination of CXCL8, IL-6 and TNF-α release. PBMC incubated (37 °C, 5% CO_2_,) for 2, 4 and 8 h were lysed to evaluate CXCL8, IL-6 and TNF-α mRNA expression.

### Apoptosis assay

Purified PBMC (10^6^ cells/ml) were cultured in an characellide A (10 µg/ml), B (10 µg/ml), Simplexide (10 µg/ml) and LPS (100 ng/ml) for 18 h. PBMC were stained with FITC-conjugated annexin V and propidium iodide (PI), according to the protocol provided by the manufacturer (Miltenyi Biotec). Quantification was performed using a MACSQuant flow cytometer (Miltenyi Biotec). Live cells were assumed to be double-negative annexin V2PI2 cells. Analysis was performed using the FlowJo v.10.

### ELISA assay

The release of CXCL8, IL-6 and TNF-α and IL-10 in the culture supernatant was measured in duplicate determinations by commercially available ELISA kits (R&D, Minneapolis, MN USA) according to the manufacturer’s instructions. The results were normalized for the number of cells per well. CXCL8 release was expressed as ng/10^6^ cells whereas IL-6 and TNF-α were expressed as pg/10^6^ cells.

#### RT-PCR

Total RNA was extracted by TRIzol reagent (Euroclone^®^, Pero, Milan, Italy) following the manufacturer’s instructions. RNA quality and integrity were estimated by spectrophotometric analysis on a Nanodrop ND-1000 spectrophotometer (Thermo Scientific, Wilmington, DE, USA). Reverse transcription was performed using the High-Capacity cDNA Reverse Transcription Kit (Applied Biosystems, Foster City CA, USA). Real-time RT-PCR was performed by means of Universal SYBR Green Supermix (Bio-Rad) on a CFX96 Real-time detection system (BioRad). *GAPDH* was used as housekeeping gene to normalize Ct (cycle threshold) values using the 2^−ΔCt^ formula. PCR efficiency and specificity were evaluated by analyzing amplification curves with serial dilutions of the template cDNA and their dissociation curves. Each cDNA sample was analyzed in triplicate and the corresponding no-RT mRNA sample was included as a negative control. The data were analyzed with iCycler iQ analysis software (Biorad) and the changes in *CXCL8*, *IL6* and *TNFα* mRNAs were expressed as 2^−ΔCt^.

### Reactive oxygen species (ROS) production

PBMC were incubated for 30 min after the addition of 10 μg/ml H2DCF-DA (Life Technologies, Milan, Italy). H2DCF-DA is a fluorogenic dye that allows the evaluation of hydroxyl peroxyl and other ROS activities within the cell. Once diffused into the cell, H2DCF-DA is deacetylated by cellular esterases to a nonfluorescent molecule, which is oxidized by ROS into 20, 70 dichlorofluorescein (DCF). This latter is highly fluorescent and can be determined by fluorescence spectroscopy with maximum excitation and emission wavelengths of 492–495 and 517–527 nm, respectively. The first group of cells was washed in phosphate buffer saline (PBS), then resuspended in RPMI supplemented with 5% of FBS and finally seeded in a 96-well precoated plate with methanol alone, characellide A or B (10 μg/ml) or stimulated with LPS (100 ng/ml). The second group of PBMC was preincubated (37 °C, 5% CO_2_, 1 h) with characellide A or B and then stimulated with LPS. Finally, immediately after LPS stimulation, the plates were placed in an EnSpire Multimode Plate Reader (Perkin Elmer). The ability of stimuli to induce cytoplasmic ROS-catalyzed oxidation of DCFH in PBMC was measured as compared to the negative control (the medium alone). The data were expressed as DCF mean fluorescence intensity (MFI) measured up to 50 min with 10 min span at an excitation wavelength of 492–495 nm and emission at 517–527 nm.

### Statistical analysis

The data are expressed as mean values ± SEM of the indicated number of experiments. Statistical analysis was performed in Prism 6 (Graph-Pad Software). Statistical analysis was performed by Student’s *t *test or one-way analysis of variance followed by Dunnett’s test (when comparison was made against a control) or Bonferroni’s test (when comparison was made between each pair of groups) by means of Analyse-it for Microsoft Excel, version 2.16 (Analyse-it Software, Ltd., Leeds, United Kingdom). Statistically significant differences were accepted when the *p *value was at least ≤ 0.05.

## Results

### Characellide-A induces the production of CXCL8, IL-6 and TNF-α from human PBMC

**Fig. 1:**
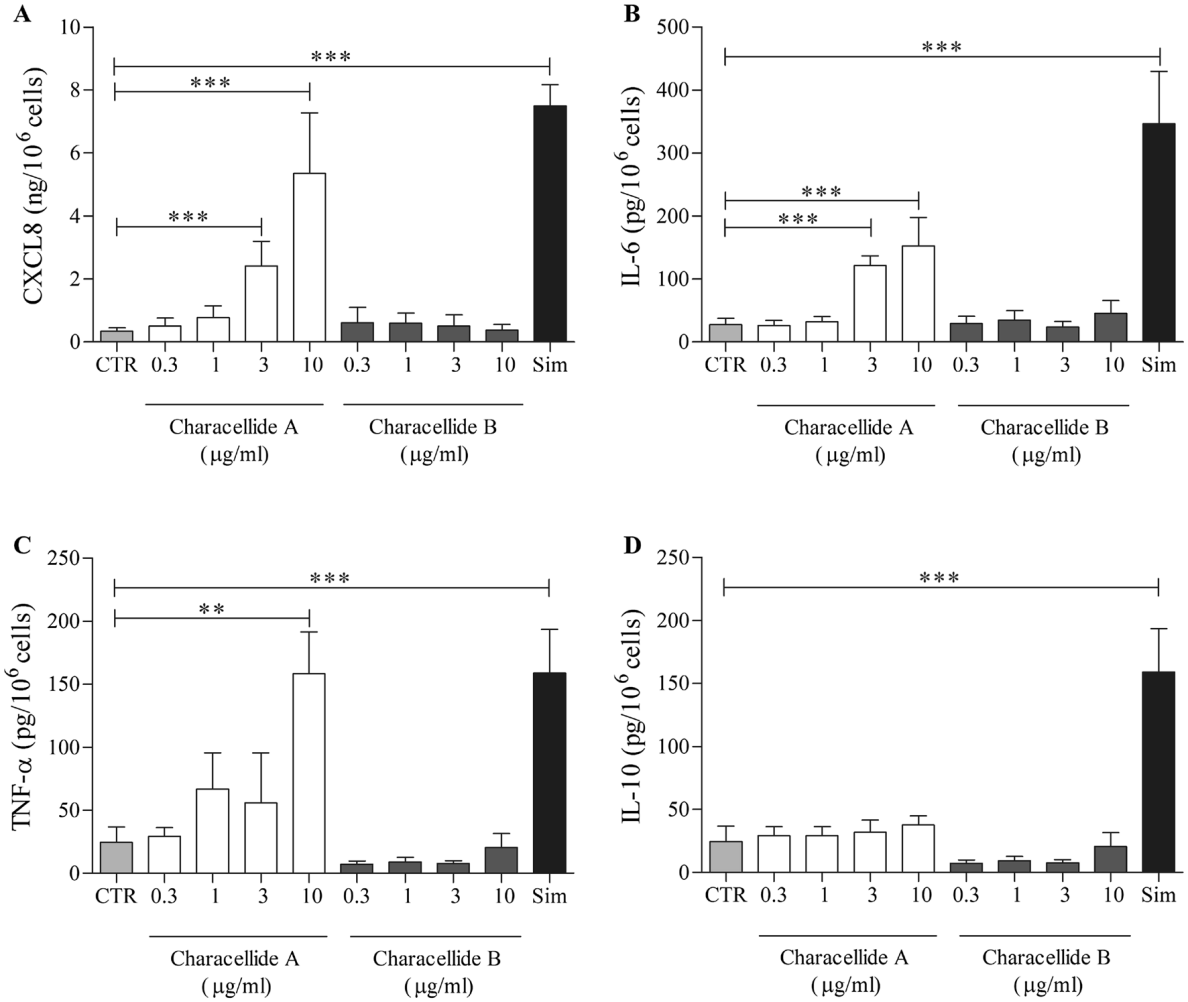
Effects of characellide A and B on CXCL8, IL-6, TNF-α and IL-10 release from peripheral blood mononuclear cells (PBMC). Polystyrene plates were coated with methanol alone or indicated concentrations of glycolipids dissolved in methanol. Solvent was air-dried immediately before the addition of cells. PBMC were incubated (37 °C, 5% CO_2_, 16 h) with medium alone (CTR), characellide A and B or 10 µg/ml simplexide (Sim). At the end of incubation, the supernatants were collected and centrifuged (300×*g*, 4 °C, 5 min). Cytokines and chemokines were determined by ELISA. The values are expressed as ng [for CXCL8 (**A**)] and pg [for IL-6 (**B**), TNF-α (**C**) and IL-10 (**D**)] per 10^6^ of total cells. Data are representative of five independent experiments obtained from different donors. *p < 0.05, **p < 0.01, ***p < 0.001

In the first part of the experiments, we evaluated the effects of characellide A and B on cytokine and chemokine release from human PBMC. The effect of compounds on cell viability was checked. Cells were incubated (37 °C, 5% CO_2_, 16 h) with increasing concentrations (0.3–10 µg/ml) of characellide A and B and the release of CXCL8, IL-6, TNF-α and IL-10 was determined by ELISA. We used the glycolipid simplexide isolated from the marine sponge *Plakortis simplex* as a positive control (Vintonyak et al. 2008). Figure 1 shows that characellide A, but not B, induced CXCL8 (Fig. 1A), IL-6 (Fig. 1B) and TNF-α (Fig. 1C) from PBMC. CXCL8 and IL-6 release was significantly increased from 3 µg/ml onwards, while TNF-α release was significant only at a concentration of 10 µg/ml. Conversely, both characellides A and B did not induce IL-10 release from human PBMC (Fig. 1D). The production of TNF-α induced by characellide A was comparable to that of simplexide (10 µg/ml), our positive control, whereas the release of IL-6 and TNF-α was lower (Fig. 1A–C). To exclude that the effect of characellide could be due to LPS contamination, a well-characterized stimulus for these cells (Loffredo et al. 2014), PBMC were stimulated with characellide in the presence of polymyxin B (50 mg/ml), a potent inactivator of LPS (Kolomaznik et al. 2018). Polymyxin B did not influence the capacity of characellide A to induce the release of CXCL8, IL-6, and TNF-α, whereas it almost completely suppressed the production of cytokines and chemokines induced by LPS (Table 1).

**Table 1:** Effect of polymixin B on characellide A- and LPS-induced release of CXCL8, IL-6 and TNF-α from PBMC

	CXCL8 (ng/10^6^ cells)	IL-6 (pg/10^6^ cells)	TNF-α (pg/10^6^ cells)
CTR	0.34 ± 0.10	27.83 ± 10.03	24.35 ± 12.34
Characellide A	5.35 ± 1.91*	152.6 ± 45.14*	158.4 ± 33.06*
LPS	35.36 ± 0.98*	4346 ± 10.45*	580.5 ± 119.3*
Polymyxin B	0.45 ± 0.60	30.47 ± 9.06	22.53 ± 10.44
Characellide A + polymyxin B	8.31 ± 2.76*	163.1 ± 15.10*	163.7 ± 15.34*
LPS + polymyxin B	10.63 ± 0.17**	240.5 ± 24.40**	55.28 ± 5.92**

PBMC were incubated (37 °C, 5% CO_2_, 16 h) with Characellide A (10 µg/ml) or LPS (100 ng/ml) either in the absence (CTR) or the presence of polymyxin B (50 µg/ml). Data are the mean ± SE of three experiments obtained from different donors

*p < 0.001 versus respective untreated (CTR)

**p < 0.001 versus LPS alone

**Fig. 2:**
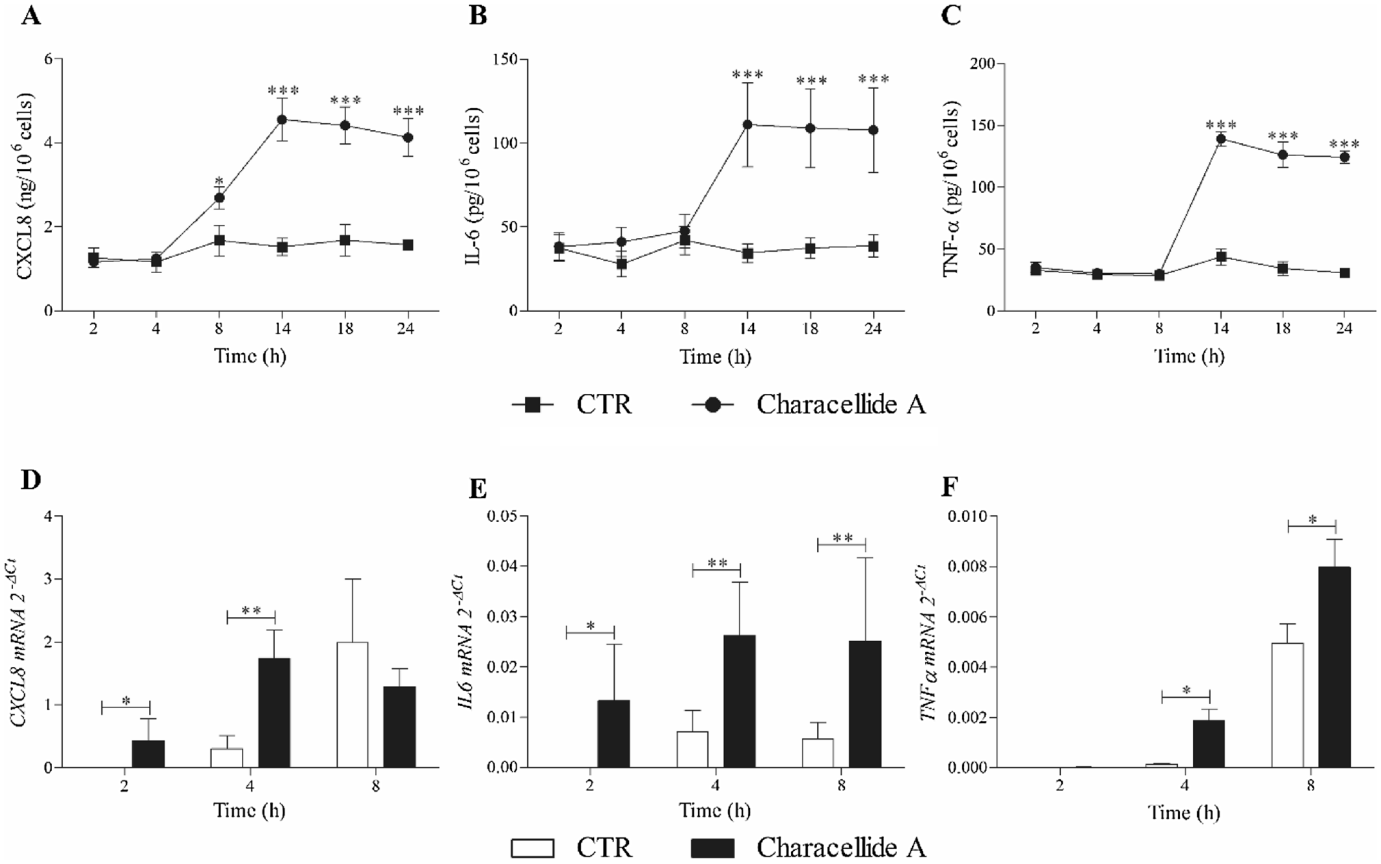
Kinetics of CXCL8, IL-6 and TNF-α release and mRNA expression induced by characellide A in PBMC. Polystyrene plates were coated with methanol alone or characellide A (10 µg/ml) dissolved in methanol. Solvent was air-dried immediately before the addition of cells. Cells were incubated (37 °C, 5% CO_2_) at indicated times with medium alone (CTR) or characellide A. At the end of incubation the supernatants were collected and centrifuged (300×*g*, 4 °C, 5 min) and the cells were lysed as described in Methods. CXCL8 (**A**), IL-6 (**B**) and TNF-α (**C**) in the supernatants were determined by ELISA. Data are the mean ± SEM of five independent experiments. *CXCL8* (**D**), *IL6* (**E**) and *TNFα* (**F**) mRNA expression was analyzed by real-time PCR (see Methods) and values were normalized to *GAPDH* and expressed as 2^−ΔCt^. Data are the mean ± SEM of five independent experiments obtained from different donors. *p < 0.05, **p < 0.01, ***p < 0.001

In the next group of experiments, we examined the kinetics of CXCL8, IL-6 and TNF-α release from PBMC stimulated with an optimal concentration of characellide A (10 µg/ml). Figure 2 reports that CXCL8 (Fig. 2A) release began after 8 h and reached a plateau after 14 h. Moreover, characellide A-induced release of IL-6 and TNF-α (Fig. 2B, C) was slower, starting at 14 h keeping a steady state up to 24 h. These distinct kinetics outcomes of characellide A on PBMC shown in Fig. 2 A-C suggest that the release of these mediators may be due to de novo gene expression rather than to secretion of preformed CXCL8, IL-6 and TNF-a from intracellular stores. Thus, we examined whether characellide A activated cytokine/chemokine gene expression in PBMC by real-time quantitative PCR. The kinetics of induction was quite different. The mRNA expression of *CXCL8* (Fig. 2D) and *IL6* (Fig. 2E) induced by characellide A were rapid and began after 2 h. In particular, *CXCL8* mRNA peaked at 4 h and declined after 8 h whereas *IL6* mRNA reached a plateau after 4 h. *TNFa* mRNA was first evident at 4 h and progressively increased up to 8 h (Fig. 2F). In support of these results, fresh PBMC did not contain preformed CXCL8, IL-6 and TNF-α (data not shown). These data indicate that characellide A induces a proinflammatory profile by cytokine/chemokine production and gene expression from human PBMC in a time-dependent manner.

### CD1d is required for PBMC activation induced by characellide A

**Fig. 3:**
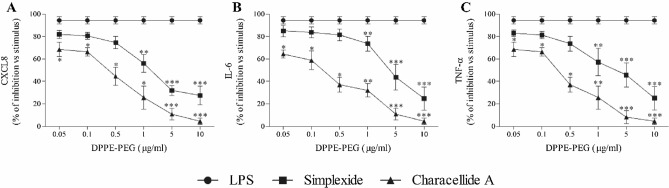
Effect of DPPE-PEG on characellide A-induced CXCL8, IL-6, and TNF-α release from PBMC. PBMC were preincubated (37 °C, 5% CO_2_, 30 min) with medium alone, or with the indicated concentrations of DPPE-PEG and then stimulated (16 h) with LPS (closed circle;100 ng/ml), simplexide (closed square;10 µg/ml) and characellide A (closed traingle;10 µg/ml). At the end of incubation the supernatants were collected and centrifuged (300×*g*, 4 °C, 5 min). CXCL8 (**A**), IL-6 (**B**) and TNF-α (**C**) in the supernatants were determined by ELISA. Data are expressed as percent of inhibition of the maximum response induced by LPS, characellide A or simplexide alone calculated as (*R* − *R*_b_)/(*R*_max_ − *R*_b_) × 100, where *R* is the release in samples treated with the agonists plus DPPE-PEG, *R*_b_ is the release in unstimulated samples and *R*_max_ is the release in samples stimulated with agonist alone. Data are the mean ± SEM of five experiments obtained from different donors. The lines represent the best fit for inhibition of LPS, characellide or simplexide. *p < 0.05, **p < 0.01, ***p < 0.001

CD1d is known to be the most important target of some metabolites isolated from marine sponges including simplexide (Loffredo et al. 2014). We hypothesized that characellide A could be involved in PBMC activation through the CD1d pathway. To evaluate whether DPPE-PEG, a CD1d antagonist, was able to inhibit the cytokine and chemokine release from human PBMC induced by characellide, we preincubated (37 °C, 5% CO_2_, 30 min) the cells with DPPE-PEG, and then seeded in precoated plates with characellide A. Figure 3 shows that DPPE-PEG (0.05–10 µg/ml) inhibited in a concentration-manner the release of CXCL8 (Fig. 3A), IL-6 (Fig. 3B) and TNF-α (Fig. 3C) induced by characellide A, from PBMC. Similar results were observed when we stimulated our positive control with simplexide (Fig. 3A–C). Conversely. DPPE-PEG had no effects on LPS-induced CXCL8, IL-6 and TNF-α release (Fig. 3 A–C) supporting the specificity of the inhibitory effect of DPPE-PEG linked to CD1d blocking.

### Characellide-A and ROS production from PBMC

**Fig. 4:**
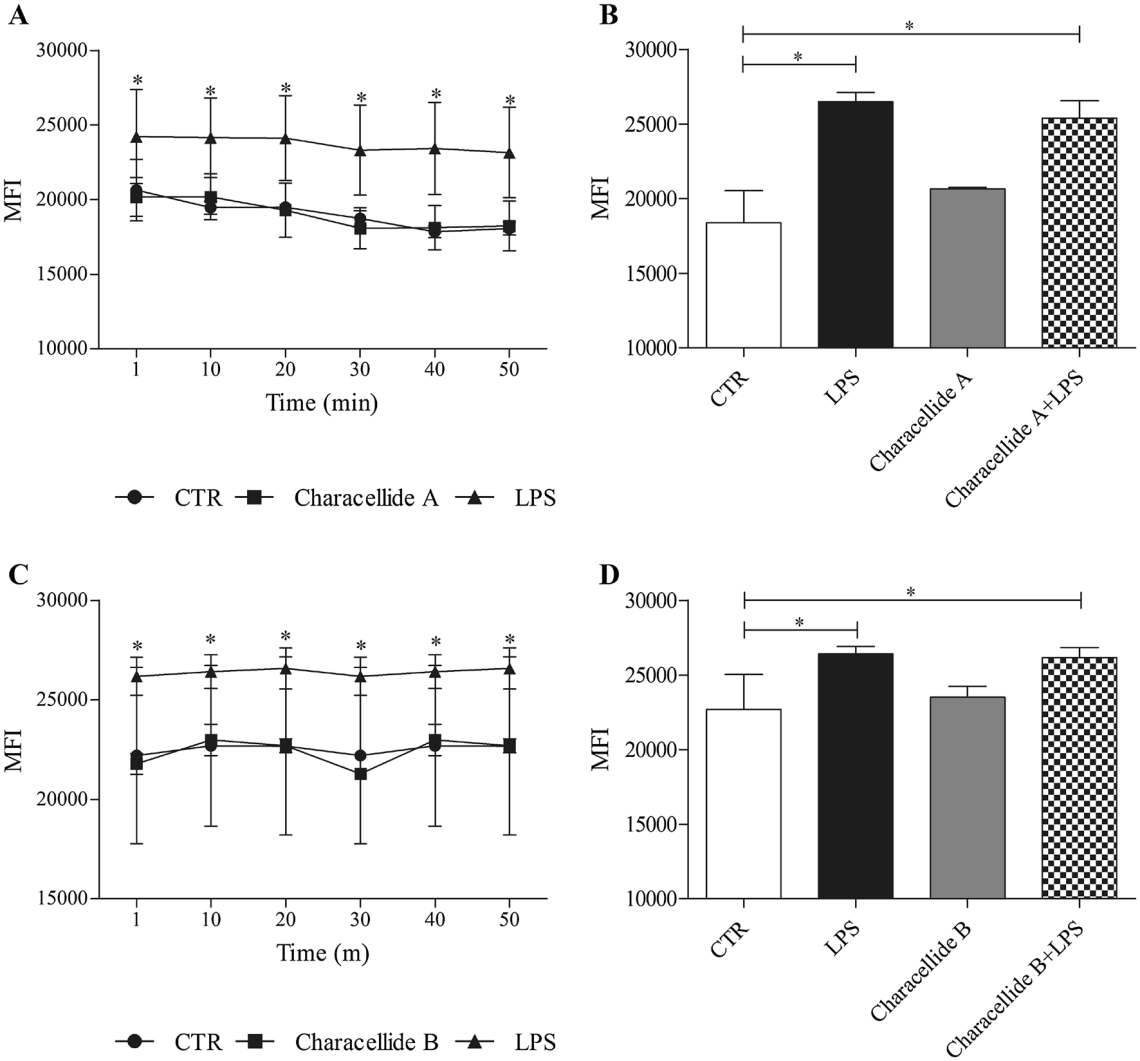
Effect of Characellide A and Characellide B on ROS production from PBMC. PBMC were preincubated (37 °C, 5% CO_2_, 30 min) with 2′,7′-dichlorodihydrofluorescein diacetate (H_2_DCFDA, 10 μM), washed, and the added to precoated plates coated with methanol alone, Characellide A (10 µg/ml) (**A**), Characellide B (10 µg/ml) (**C**), or stimulated with LPS alone (**A, C**). In panels **B** and **D**, cells were preincubated (1 h) with Characellide A and Characellide B then stimulated with LPS. Immediately after the stimulation, the cells were placed in a multimode microplate reader (EnSpire Multimode Plate reader, PerkinElmer), and DCF fluorescence intensity was quantitatively measured for 50 min (**A, C**) or at 10 min (**B, D**). The results were expressed as mean fluorescence intensity (mean ± SEM of five independent experiments obtained from different donors).*p < 0.05

Characellide A and B have been shown to inhibit ROS production from microglia cells (Afoullouss et al. 2019). Based on this study, we first investigated the effect of characellide A, B and LPS on ROS production in human PBMC. Figure 4A, C illustrates the kinetics profile (up to 50 min) of the production of ROS induced by the control medium, characellide A and B or LPS. LPS induced ROS production by PBMC whereas, in contrast, characellide A and B had no effect. To evaluate whether characellide A and B were able to inhibit ROS production induced by LPS, we preincubated (37 °C, 5% CO_2_, 1 h) cells with characellide A or B and then stimulated (10 min) them with LPS. Figure 4B, D demonstrates that LPS-induced ROS production in PBMC was not influenced by characellide A and B stimulation.

## Discussion

In this study, the marine glycolipopetide characellide A was shown to induce the expression and release of cytokines and chemokines from human peripheral blood mononuclear cells. A is stronger in inducing CXCL8 production than IL-6 and TNF-α. This compound exerts comparable effects to simplexide, a simpler glycolipid also isolated from a marine sponge, in releasing CXCL8 and TNF-α (Loffredo et al. 2014). Moreover, the stimulatory activity of characellide A is not inhibited by polymyxin B suggesting that it is really due to the natural molecule rather than traces of lipopolysaccharide, a well-known PBMC stimulus (Jansky et al. 2003; Loffredo et al. 2014). The effects of characellide A on PBMC are mediated by CD1d expression on human PBMC.

Cellular activation could induce the release of preformed and/or de novo synthesized mediators. The release of CXCL8, IL-6 and TNF-α induced by characellide A is time-dependent requiring at least 8 h of stimulation. Therefore, the effects of characellide A are mediated by the induction of gene transcription. Indeed, characellide A activates the expression of *CXCL8, IL6* and *TNFα* but not *IL-10* mRNA, a gene which transcription is induced by simplexide in monocytes (Loffredo et al. 2014). However, a small albeit not significant release of IL-10 is induced by characellide A. Moreover, fresh lysated PBMC did not contain preformed CXCL8, IL-6, TNF-α and IL-10 supporting the evidence that the production of these mediators is only mediated by gene transcription.

The profile of cytokines/chemokines induced by characellide A in PBMC suggests that this compound may potentially exert both proinflammatory and immunoregulatory activities. Both CXCL8 and TNF-α are potent inflammation molecules and are involved in recruitment and activation of inflammatory cells (Turner et al. 2014). On the other hand, IL-6 is a regulatory mediator (Tanaka et al. 2014) that can at least partially unfold the immunosuppressive function previously demonstrated by characellide A on BV-2 microglia cell activation (Afoullouss et al. 2019). In particular, characellide A inhibited ROS production in BV-2 microglia cells induced by LPS (Afoullouss et al. 2019). Here, we have repeated the same experiments on PBMC obtaining different results. LPS induced ROS production by PBMC but this effect is not inhibited by characellide A indicating that this molecule exerts a different effect depending on the cell. Interestingly, the inhibition of ROS production in LPS-stimulated microglia cells was induced by both characellide A and B (Afoullouss et al. 2019). By contrast, here we demonstrated that ROS production in LPS-stimulated PBMC was not inhibited by both characellide A and B. The difference in bioactivity for both characellides is intriguing as they are epimers and only differ from the configuration at one chiral center of the sugar unit. The 2-amino-2-deoxyglucuronamide of characellide A is replaced by a 2-amino-2-deoxygalacturonamide for characellide B. The *S* configuration at position C-4 of the sugar unit seems to be an important structural feature to enhance the bioactivity.

In fact, unlike the results on microglia cells, our results support the hypothesis that the structural differences between characellide A and B are enough to have a different activatory effect on PBMC. Converse to characellide A, characellide B was unable to activate the cytokine and chemokine production by PBMC at any concentration tested.

Many natural molecules are modulators of innate and adaptive immunity (Fujii et al. 2006; Loffredo et al. 2014; Amèlie Roux et al. 2019). Some metabolites extracted from marine sponges, such as α-GalCer and simplexide, function as antigens primarily by interacting with CD1d expressed on immune cells including monocytes and lymphocytes (Berntman et al. 2005; Patel et al. 2011; Loffredo et al. 2014). We found that characellide A activity, akin to simplexide activity, is dependent on the interaction with CD1d because it is completely inhibited by a potent CD1d antagonist.

Canonically, the presentation of CD1d-bound molecules to natural killer T (NKT) cells activates cytokine/chemokine production in both NKT cells and subsequently in antigen-presenting cells (Gordon and Galli 1990; Barral and Brenner 2007; Venkataswamy et al. 2009; Zhang et al. 2011). Previously we demonstrated that simplexide can itself generate intracellular signals directly by interaction with CD1d. Therefore the presence of NKT cells was not mandatory for simplexide-induced cytokine production from monocytes (Loffredo et al. 2014). A limit of our study is that it is conducted on PBMC. PBMC are any peripheral blood cell having a round nucleus. These cells consist of lymphocytes (T and B cells, NK cells) and monocytes, therefore, a question that remains to be answered is which cells belonging to PBMC are directly or indirectly activated by characellide A. It will be interesting to assess the effect of characellide A on purified cell populations of PBMC, in particular monocytes, to better understand its cellular activation mechanism.

In conclusion, characellide A is a novel and potent stimulus for cytokine and chemokine production in human PBMC. Our data suggest that this molecule behaves as a “danger” signal and may be involved in the activation of adaptative/innate immune responses. Further studies with purified cells are required to fully understand biological activity of characellide.

Since some of the sponge-derived metabolites were included in clinical and preclinical trials showing their efficacy as modulators of immune response (Gunasekera et al. 1989; Alcaraz and Paya 2006; Fattorusso and Taglialatela-Scafati 2009; Sagar et al. 2010; Bibi et al. 2017; Negi et al. 2017; Karpinski 2019; Yurchenko et al. 2020), the observation that characellide A is a potent in vitro activator of PBMC raising interesting perspectives on its potential pharmacological application.

## Abbreviations

α-GalCer: α-Galactosylceramide
CD1d: Cluster of differentiation-1d
CXCL8: C–X–C motif ligand 8
DCF: 20,70-Dichlorofluorescein
DPPE-PEG: 1,2-Bis(diphenylphosphino)ethane-polyethylene glycolmonomethylether
GAPDH: Glyceraldehyde 3-phosphate dehydrogenase
H_2_DCFDA: 2′,7′-Dichlorodihydrofluorescein diacetate
IFN-γ: Interferon-γ
IL-4: Interleukin-4
IL-6: Interleukin-6
IL-10: Interleukin-10
iNKT: Invariant natural killer T cells
LPS: Lipopolysaccharide
PBMC: Peripheral blood mononuclear cells
ROS: Reactive oxygen species
TFA: Trifluoroacetic acid
TNF-α: Tumor necrosis factor-α
